# Hydrogen isotopes retention studies using laser and microwave induced plasma coupling

**DOI:** 10.1038/s41598-025-96546-x

**Published:** 2025-04-12

**Authors:** N. Vujadinovic, I. Traparic, B. D. Stankov, D. Rankovic, M. Kuzmanovic, M. Ivkovic

**Affiliations:** 1 Institute of Physics, University of Belgrade, 11080 Belgrade, Serbia; 2 Faculty of Physical Chemistry, University of Belgrade, 11158 Belgrade, Serbia; 3 Present Address: Vinca Institute of Nuclear Science, University of Belgrade, 11000 Belgrade, Serbia

**Keywords:** Hydrogen isotopes retention, Laser ablation, Laser induced desorption, Microwave induced plasma, Plasma-facing components, LIBS, Laser-produced plasmas, Chemical physics

## Abstract

The detection of deuterium and tritium retention in fusion devices via optical emission spectroscopy (OES) faces significant challenges due to experimental limitations, particularly in resolving hydrogen isotope Balmer alpha lines (H_α_, D_α_, and T_α_). In this study, we propose and evaluate the coupling of laser ablation and laser-induced desorption with microwave-induced plasma (MIP) as an approach to resolve this problem. This approach effectively meets the resolution requirements for Balmer alpha lines, overcoming limitations of standard laser-induced breakdown spectroscopy (LIBS) setups. Optimization of Nd:YAG laser ablation was performed using pure copper and tungsten targets, while desorption, including femtosecond (fs) laser-induced desorption, was studied on graphite powder mixed with heavy water and water. The results demonstrate a significant improvement in spectral resolution and analytical performances, highlighting the potential of this technique for tritium retention studies in plasma-facing components.

## Introduction

Diagnostics of the fusion plasma reactors are critical for ensuring their safe and proper stable operation. Among these diagnostics, the hydrogen isotope retention, particularly tritium, in plasma facing components (PFC) are probably the most important ones^1^. Techniques such as ion beam analysis (IBA) and thermal desorption spectroscopy studies (TDS) are highly reliable and commonly used PFC diagnostics methods^2–5^. However, these methods are constrained to laboratory settings and require complex equipment. Consequently, sections of the PFC or test targets positioned on various places within the vacuum vessel must be demounted from the reactor wall^6^ to be analyzed.

To enable in-situ analysis of PFC, laser induced breakdown spectroscopy (LIBS) is used as an effective solution to overcome limitations of traditional methods. LIBS is a minimally invasive, non-contact technique suitable for multi-element analysis, including depth profiling, without requiring sample preparation. The technique is adaptable for vacuum or low-pressure gas environments and has been applied across diverse fields, such as nitrogen detection in soil^7^, explosives detection^8^, olive oil classification^9^, cadmium detection in drinking water^10^, and even the identification of malaria biomarkers^11^, bacteria^12^ or SARS^13^. LIBS is also commonly used for analyzing metal purity, alloys, jewelry^14^, archaeological and other samples. Reviews^15–18^ and recent studies^19–21^ provide comprehensive insights into the advancements of LIBS for fusion applications, particularly its potential for in-situ diagnostics.

The most important application of LIBS for plasma fusion reactor wall diagnostics is the study of hydrogen isotope retention, which relies on measurement of their Balmer alpha spectral lines. A significant challenge in this application is resolving the closely spaced lines caused by the small isotope shift. Even high-resolution spectrometers struggle to resolve these lines, due to significant Stark broadening under standard LIBS plasma conditions^22^. Partial resolving of a hydrogen and deuterium Balmer alpha lines (with isotope shift of 0.18 nm) has been achieved in studies using double-pulsed LIBS, where line fitting with a Voigt function was employed^17,23,24^. More recently, approaches based on femtosecond (fs) laser ablation^25^ and fs LIBS^26^ have been applied to hydrogen isotope retention diagnostics, demonstrating further advancements in this field^27–32^.

The use of the TEA CO_2_ lasers or Nd:YAG in He^33–35^ or filament fs laser LIBS^27,30^ demonstrates the possibility of LIBS to resolve H_α_ and D_α_ lines. However, resolving T_α_ lines presents a greater challenge due to the stricter requirements for low electron density^22^. In this study, we propose overcoming these challenges by coupling laser and microwave-induced plasma (MIP) to achieve the necessary plasma conditions. Two sample introduction methods are employed: laser ablation using an Nd:YAG laser and fs laser-induced desorption, both integrated with microwave-induced low-pressure plasma.

## Experiment

Microwave induced plasma is the primary method used in this research for excitation and resolution of hydrogen isotope lines. The combination of MIP and laser induced plasma was used earlier for the enhancement of the LIBS^36,37^, where the addition of microwaves increased electron density, temperature, and plasma duration and dimensions. However, this enhancement also increased Stark broadening, making the method unsuitable for tritium retention studies. In contrast, MIP source operating at atmospheric pressure has plasma parameters^38,39^ suitable for resolving D_α_ and T_α_ spectral lines, as analyzed in^22^. At low gas pressures, MIP achieves even smaller electron densities^40^, minimizing Stark and Van der Waals broadening, making them negligible in comparison with other broadening mechanisms (Doppler and instrumental). For this study, a Beenakker resonator cavity with an 8 mm diameter and 14 cm long capillary tube was used, with an optical window and evacuation port mounted at the end. MIP was generated using an AHF Analysen Technik GMW 24–301 DR 2.45 GHz microwave generator with a maximum power of 100 W. A double gauge gas regulator and needle valve regulated gas flow withing the tube. Gas pressure was measured with a manometer, and a mechanical vacuum pump was used for evacuation and maintaining stable argon gas flow.

**Fig. 1:**
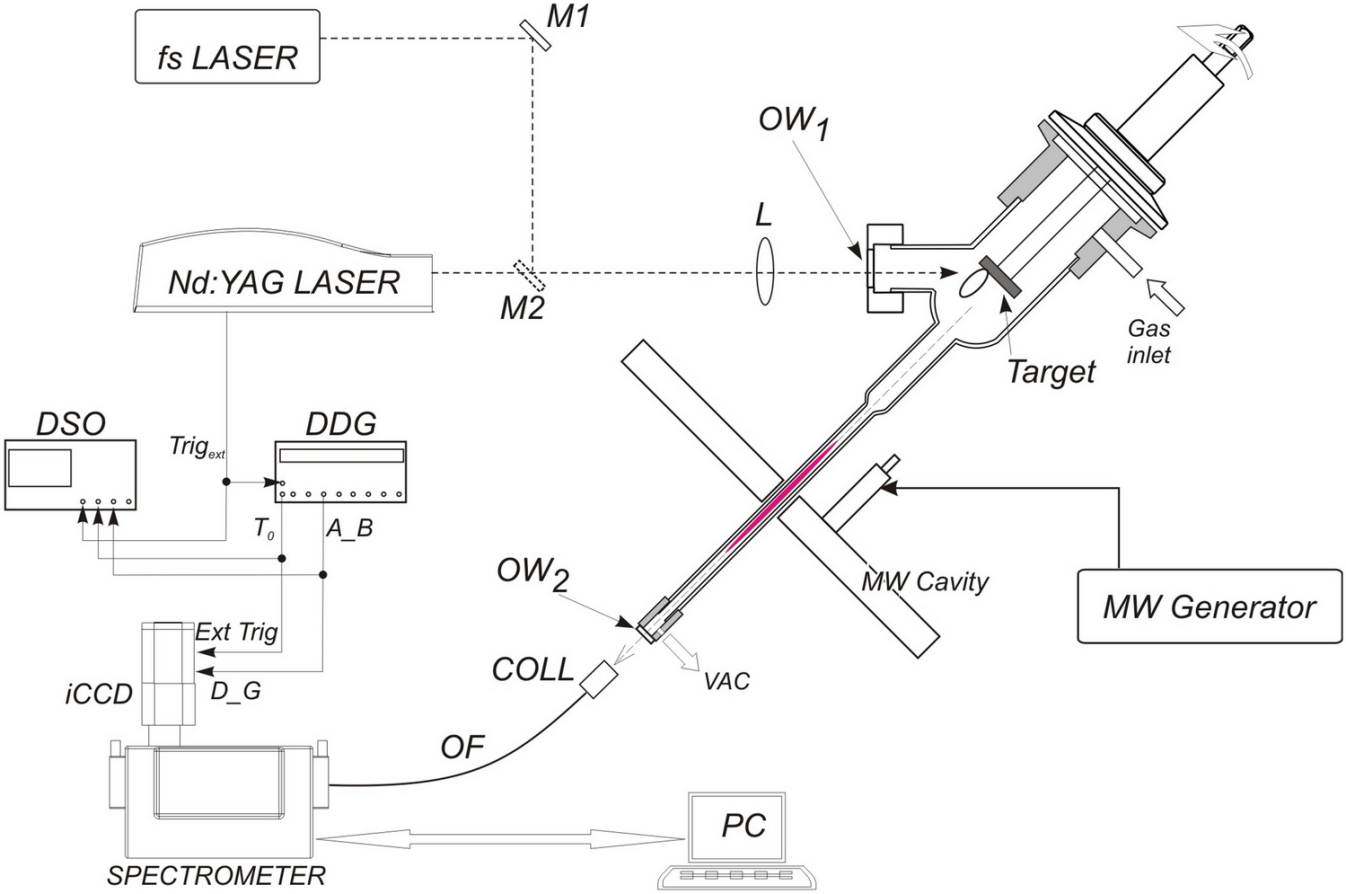
Experimental setup for laser ablation (using Nd:YAG) and laser induced desorption (using fs Yb:YAG laser) as methods for sample introduction in microwave induced plasma (MIP). DSO—digital storage oscilloscope, DDG—digital delay generator, COLL—collimator of the emitted radiation into the fiber, OF—optical fiber, M—folding mirrors, VAC—vacuuming port, L—focusing lens, OW—optical windows.

The first method used for sample introduction was laser ablation, a widely used technique in analytical spectroscopy for introducing samples into excitation sources such as inductively coupled plasma (ICP), MIP, LIBS or mass spectrometry^41–43^. For this purpose, we used a laboratory made laser ablation cell, see Fig. 1. This cell was constructed as an elongation of the capillary tube thus enabling the most efficient transport of the ablated material into the MIP. The target was placed in a custom built holder with vacuum feedthrough, allowing rotation to expose fresh target surface area to the laser beam. Ablation was performed using Quantel 450 Nd:YAG (1064 nm, 6 ns pulse duration, 10 Hz maximum repetition rate, 450 mJ maximum energy). Laser beam was focused with a *f* = 12.5 cm lens through the window and onto the target. Special cell design enables irradiation of the target at approximately 45 degrees. Small variation of incident angle enables irradiation at a variable distance from the center of the target. That way, laser induced plasma radiation, which always propagates normal to the target surface, does not reach detection system and enables recording of the radiation coming from the MIP only.

The emitted light from the MIP was collected through an optical window at the end of the capillary using a collimator (COLL) and guided via fiber optic cable (OF) either to medium resolution spectrometer Andor Shamrock 303i (with grating 1200 g/mm) or high-resolution spectrometer SOL instruments MS7504i spectrometer (with grating 1800 g/mm). The Andor spectrometer was equipped with Andor iStar DH720 - 18 F- 63 ICCD camera (256 × 1024 pixels, 26 μm pixel size), while SOL instruments spectrometer was equipped with Andor iStar DH734 - 18 F- 63 ICCD camera (1024 × 1024 pixels, 13 μm pixel size), that were used as detectors. Delay and gating of cameras were controlled with external digital delay generator (DDG, Stanford Research SRS 535), which was triggered with the signal for opening of a Nd:YAG laser Q switch. It should be noted that the separation of the optical emission signals created by LIBS and by MIP can also be achieved by changing the delay and gate time of the camera exposure.

The Nd:YAG laser, commonly used for plasma creation, is known to ablate a thick layer of material, making it unsuitable for analyzing thin films or surface-bound elements. For detecting hydrogen isotopes within thin surface layers, laser-induced desorption (LID)^44–46^, often paired with quadrupole mass spectrometry (LID-QMS)^47^, is a more suitable approach. To test the feasibility of using MIP as an alternative to the more complex QMS, a femtosecond Yb:YAG laser (Solar FX200, 1030 nm, 150 fs pulse duration, 105 nJ peak energy, 71 MHz repetition rate, 7 W average power) was employed for laser-induced desorption of hydrogen isotopes. In this setup, the detection system was triggered by the camera’s internal trigger with a variable exposure time. While this study utilized the femtosecond laser, laser-induced desorption can also be achieved with other lasers capable of heating the target without causing significant ablation.

The selected targets for these studies included a copper target for the experiment optimization in terms of gas pressure, delay time, microwave power and laser energy. A tungsten target was then introduced to verify the optimized conditions for resolving Balmer alpha spectral lines. Finally, a pill composed of graphite powder mixed with water and heavy water (D_2_O), was prepared using a hydraulic press, as previously described in^22^. This pill was tested for both laser-induced desorption and vacuum-induced desorption.

## Results

In the investigation of MIP for hydrogen isotope detection, the initial task involved optimizing the transport and excitation of sample components. Due to the challenges associated with tritium’s radioactivity, most previous research has focused on deuterated samples as a safer alternative. In this study, tungsten (W) samples containing incorporated deuterium were analyzed using laser ablation as the method for introducing samples into the MIP.

### Laser ablation

The investigation of Nd:YAG laser ablation as a method for introducing tungsten samples with incorporated deuterium into the MIP proved nearly impossible with our experimental setup. This was due to several factors: The high reflectivity of the polished samples, the shallow retention of the deuterium and high laser ablation rate. As a result, the application of MIP for hydrogen isotopes detection using laser ablation was limited to optimizing parameters for resolving hydrogen isotope Balmer alpha lines. According to^22^, D_α_ and T_α_ spectral lines can be resolved only if full width at half maximum (FWHM) of lines is less than 0.056 or even 0.027 nm, depending on their intensity ratio (1:1 or 1:10, respectively). Furthermore, FWHMs of the neighbor spectral lines must also be smaller than the wavelength separation between them and hydrogen isotope lines.

To obtain the best resolving results, using laser ablation, the signal to noise ratio (i.e., line intensities) has to be maximized by optimizing several experimental parameters, while keeping FWHM as minimal as possible. For MIP operation, microwave power and gas pressure are the most important parameters. The line intensities increase with microwave power, but the reflected power also increases. If the reflected power exceeds 15 W, there is a risk of damaging the microwave generator or overheating the discharge tube. Optimal gas pressure, which corresponds to the flow rate, must also be determined, as it dictates the time the sample remains within the MIP resonator cavity for excitation.

Additionally, the dependence of spectral line intensities on laser energy was analyzed, as laser energy influences the ablation process, specifically the ablated mass and particle dimensions. Although higher energy increases the ablated mass, it is important to assess whether larger particle dimensions might affect MIP performance by altering microwave coupling to the plasma or causing particle deposition on the tube walls.

#### Optimization of gas pressure

**Fig. 2:**
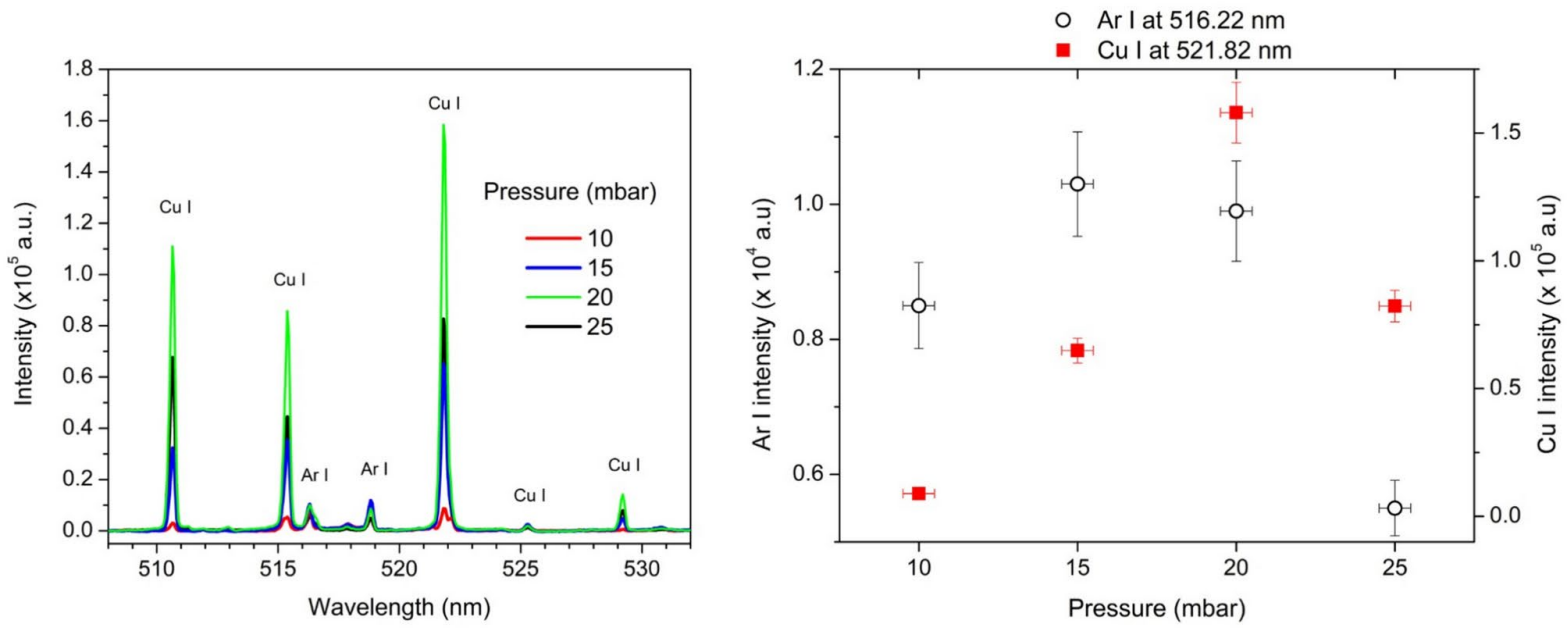
(**a**) MIP spectra of a laser-ablated Cu target in Ar at various pressures. Experimental parameters: MIP power: 75 W, delay: 8 ms, gate: 10 ms, number of accumulations: 20. (**b**) Selected Ar and Cu line intensities as a function of pressure.

The optimal gas pressure range for stable MIP operation was determined by analyzing the maximal intensity of the Ar I line at 516.22 nm (3s^2^3p^5^(^2^P^0^₃/₂)4p → 3s^2^3p^5^(^2^P^0^₃/₂)6d), as shown in Fig. 2a. The Ar I line was used to establish the optimal gas pressure range since its intensity is independent of the camera recording delay. In contrast, the intensities of the target lines, such as Cu I, depend on the gas flow rate, which determines when the ablated material reaches the plasma.

From Fig. 2b, the optimal gas pressure range for stable MIP operation was between 15 and 20 mbar.

The optimal gas pressure can be determined using the most intense spectral line, the Cu I line at 521.82 nm (3d^10^4p → 3d^10^4d). Since this line is susceptible to self-absorption, its optical thickness was evaluated. The Cu I lines at 515.32 nm (λ_1_) and 521.82 nm (λ_2_) belong to the same multiplet (transition 3d^10^4p → 3d^10^4d) as shown in Fig. 2a. To assess self-absorption of the 521.82 nm line, the intensity ratio *R* = *I *_*λ1*_/*I *_*λ2*_ was compared at various pressures to the theoretical value of *R* = 0.53 (Table 1)^48^. Results indicated no significant self-absorption for all pressures except at 10 mbar, where the lines exhibited low intensity.

**Table 1:** Experimental ratios of the intensities of Cu I spectral lines at various pressures.

Pressure (mbar)	*R* = *I *_*λ1*_/*I *_*λ2*_ (exp.)
10	0.66
15	0.54
20	0.53
25	0.54

Since the Cu I line has a significantly higher intensity at 20 mbar, see Fig. 2b, this line was used in optimization of several experimental parameters in all further investigations.

#### Optimization of material transport to the MIP

**Fig. 3:**
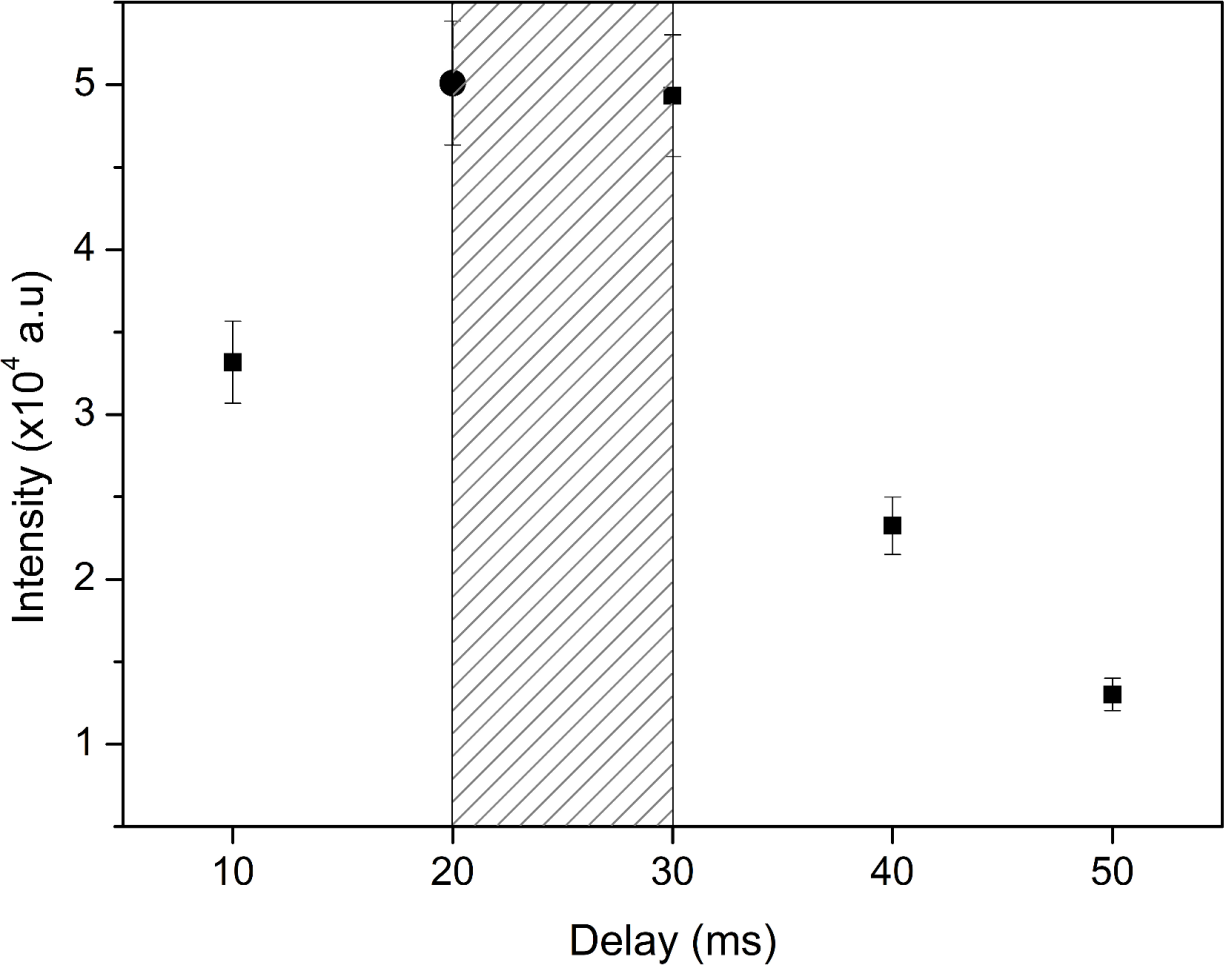
Optimization of the delay time between the laser pulse and camera triggering by maximizing intensity of the 521.82 nm Cu I line. Shaded area represents the gate time.

Gas pressure and flow regulate the duration for which the ablated material remains in the discharge, thereby influencing the recording parameters (delay and gate times). The delay corresponds to the time required for the material to travel from the target to the resonator cavity, while the gate time determines the duration the material spends in the discharge zone. An analysis of the optimal delay time is presented in Fig. 3. It should be noted that for this and all further analyses the gate was fixed to 10 ms.

Based on Fig. 3 it was concluded that the delay time should be 20 ms and that the 10 ms gate time is appropriate. During these measurements, microwave power was set to 75 W and laser energy was 250 mJ. The final spectrum was the accumulated spectrum of 20 laser shots.

#### Optimization of ablated material quantity

**Fig. 4:**
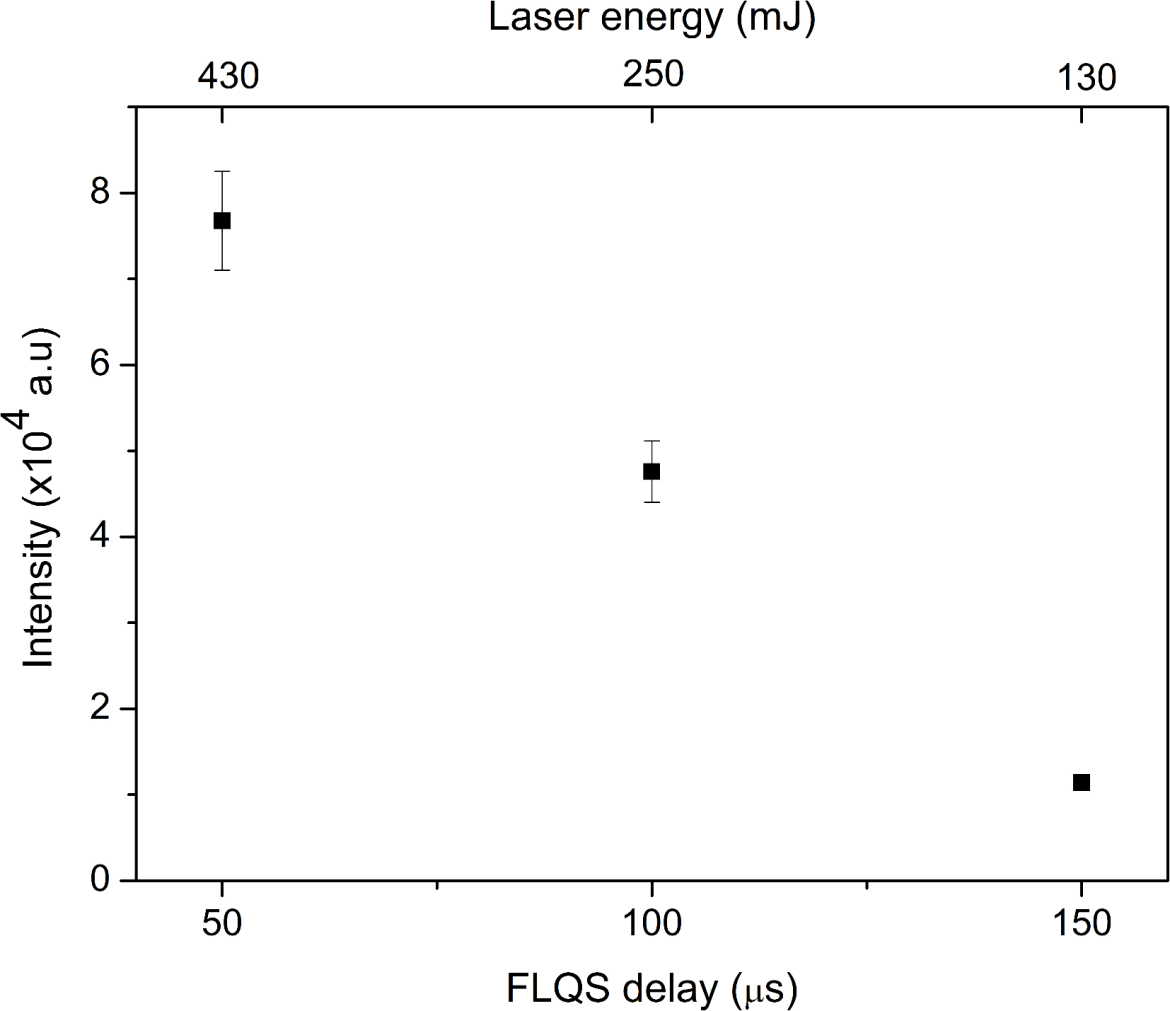
Dependence of the 521.82 nm Cu I line intensity on the laser energy controlled with FLQS delay, for the optimal pressure, delay and gate time.

The amount of ablated material is directly influenced by the laser energy used. To optimize this parameter, the laser energy was adjusted by varying the delay between the triggering of the flash lamps and the opening of the laser Q switch (FLQS). Three energy values were tested: 130, 250 and 430 mJ. The resulting graph is shown in Fig. 4.

With the increase of the laser energy, the intensity of the Cu I line also increases. This is primarily due to the ablation rate, as when the energy of the laser is higher, the ablation rate is also higher, and more material is entering the discharge region. Here, the energy of 250 mJ was chosen, as for the higher energy, the mass of the incoming material was too large, which caused the MIP discharge to shut down.

#### Selection of microwave generator power

**Fig. 5:**
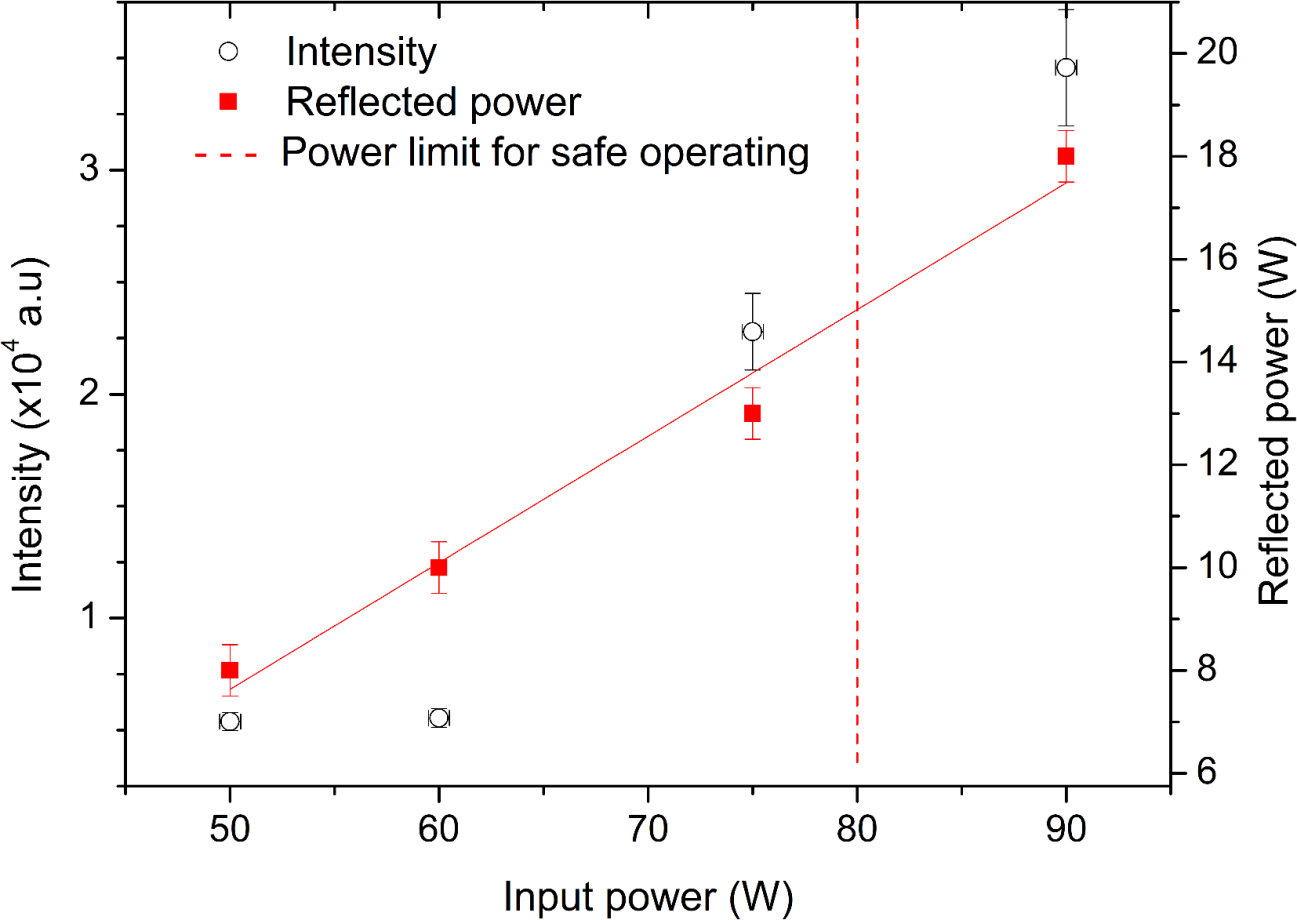
Intensity of the 521.82 nm Cu I line and microwave generator reflected power as a function of microwave generator input power.

Dependence of the line intensity on the microwave power supplied to the cavity (for optimal pressure, delay, gate and laser energy) was analyzed. Here, four powers were considered (50, 60, 75 and 90 W). Besides supplied power, the reflected power was also measured. The obtained dependance on the supplied power is shown in Fig. 5.

The results indicate that increasing the supplied power leads to a corresponding increase in line intensity. However, the reflected power also rises with higher input power. In Fig. 5, a dashed vertical line marks the input power at which the reflected power reaches 15 W. Since exceeding this threshold could overheat the source, it is not advisable to operate beyond this limit. Consequently, 75 W was selected as the optimal power setting to ensure safe and reliable generator operation.

#### Spectral resolution requirements for hydrogen isotopes retention studies

After optimizing experimental parameters for laser ablation and MIP operation the potential of this setup for hydrogen isotopes retention studies was analyzed. For such analysis, the FWHM of Balmer alpha lines should be less than 0.027 nm if one wants to detect small amounts of tritium in the first wall of future fusion reactors. The first step in this direction was to assess the instrument broadening on the spectral lines’ widths. Given the negligible Stark broadening at low MIP gas pressures (*Ne* ~ 10^12^ cm^−3^), and the electron temperature between 2000 and 3000 K, we can safely assume that the major influence in the line broadening comes from the Doppler and instrument broadening. To determine the instrumental FWHM of device, the recorded Cu I line at 521.82 nm from Fig. 2 was fitted with a Gaussian function. The resulting FWHM was 0.27 nm, which greatly exceeds the goal of having lines as narrow as 0.027 nm. This result is reasonable, considering that medium resolution Shamrock 303 imaging spectrograph with the entrance slit width of 50 µm equipped with Andor iStar DH720 ICCD camera was used for these measurements.

To reduce the instrumental broadening of the lines, the high resolution MS7504i spectrometer with the entrance slit width of 30 µm equipped with Andor iStar DH734 - 18 F- 63 ICCD camera was used for recording spectral lines of tungsten. As part of the optimization of the optical system (selection of the spectrometer and slit width), W target was used to verify whether the W line FWHM is less than 0.04 nm, which corresponds to the wavelength separation between hydrogen Balmer alpha line at 656.28 nm and W I line at 656.32 nm.

**Fig. 6:**
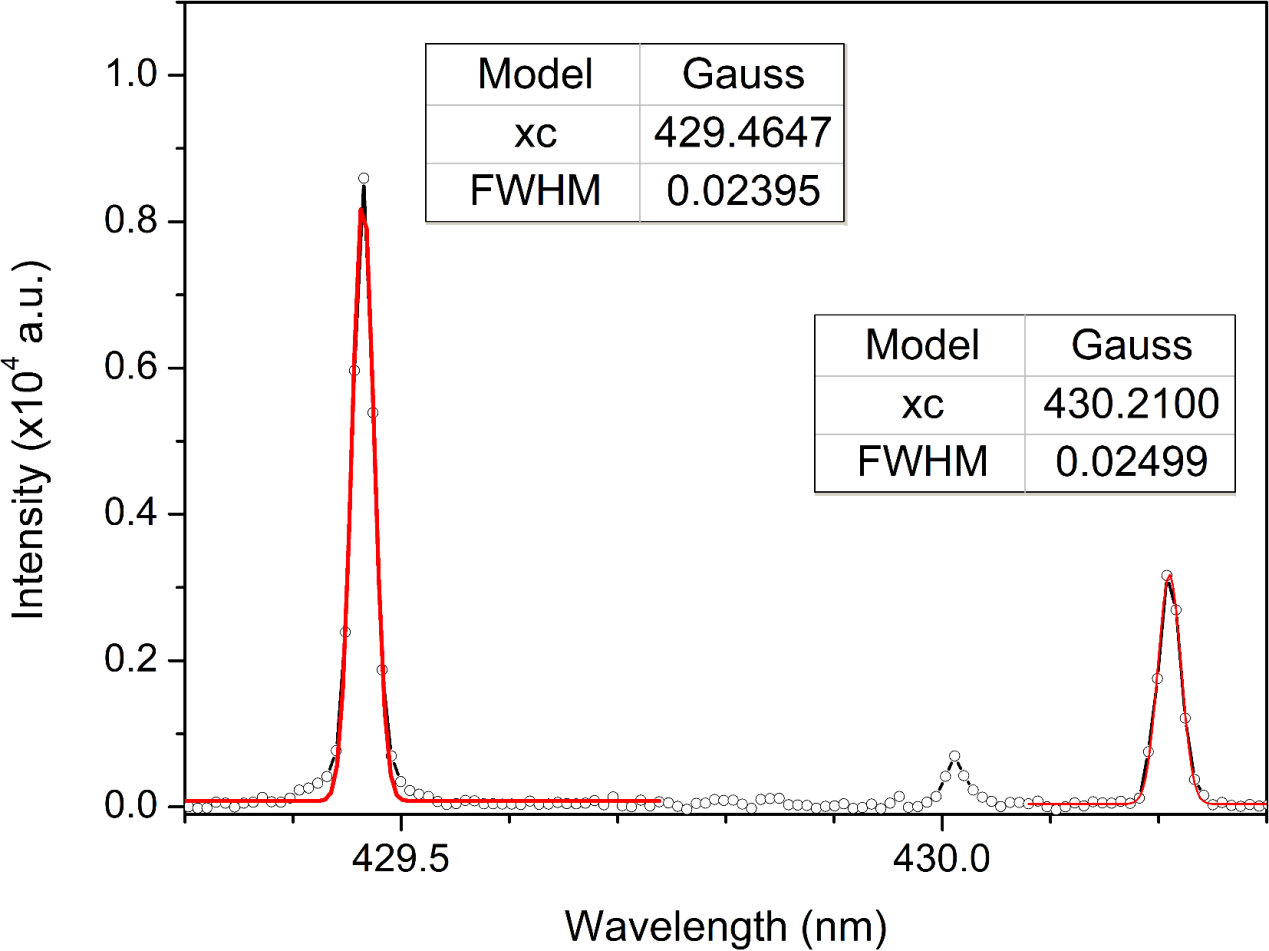
Gaussian fit of W I lines recorded with high resolution spectrometer with a 30 µm entrance slit width.

Gaussian fitting of the W I lines (Fig. 6) showed the FWHM of 0.024 nm at 429.46 nm and 0.025 nm at 430.21 nm, meeting the required resolution. This demonstrates that the setup is capable of enabling precise determination of the H_α_ line intensity.

### Desorption as a method for sample introduction in MIP

To obtain the H_α_ and D_α_ FWHM values and to test whether resolving the D_α_ and T_α_ lines is possible in this configuration, and for previously determined optimal experimental parameters of 20 ms delay and 10 ms gate time, 75 W MIP input power and a 30 µm entrance slit width of the high resolution spectrometer, a mixture of graphite powder, heavy water and water, pressed into a pill, was used as the target. In section"Desorption due to vacuuming", the results of the MIP spectrum for desorption induced by the vacuuming alone are presented, while in section"Laser induced desorption", the results of desorption induced by fs laser heating in combination with the vacuum pump are presented.

#### Desorption due to vacuuming

**Fig. 7:**
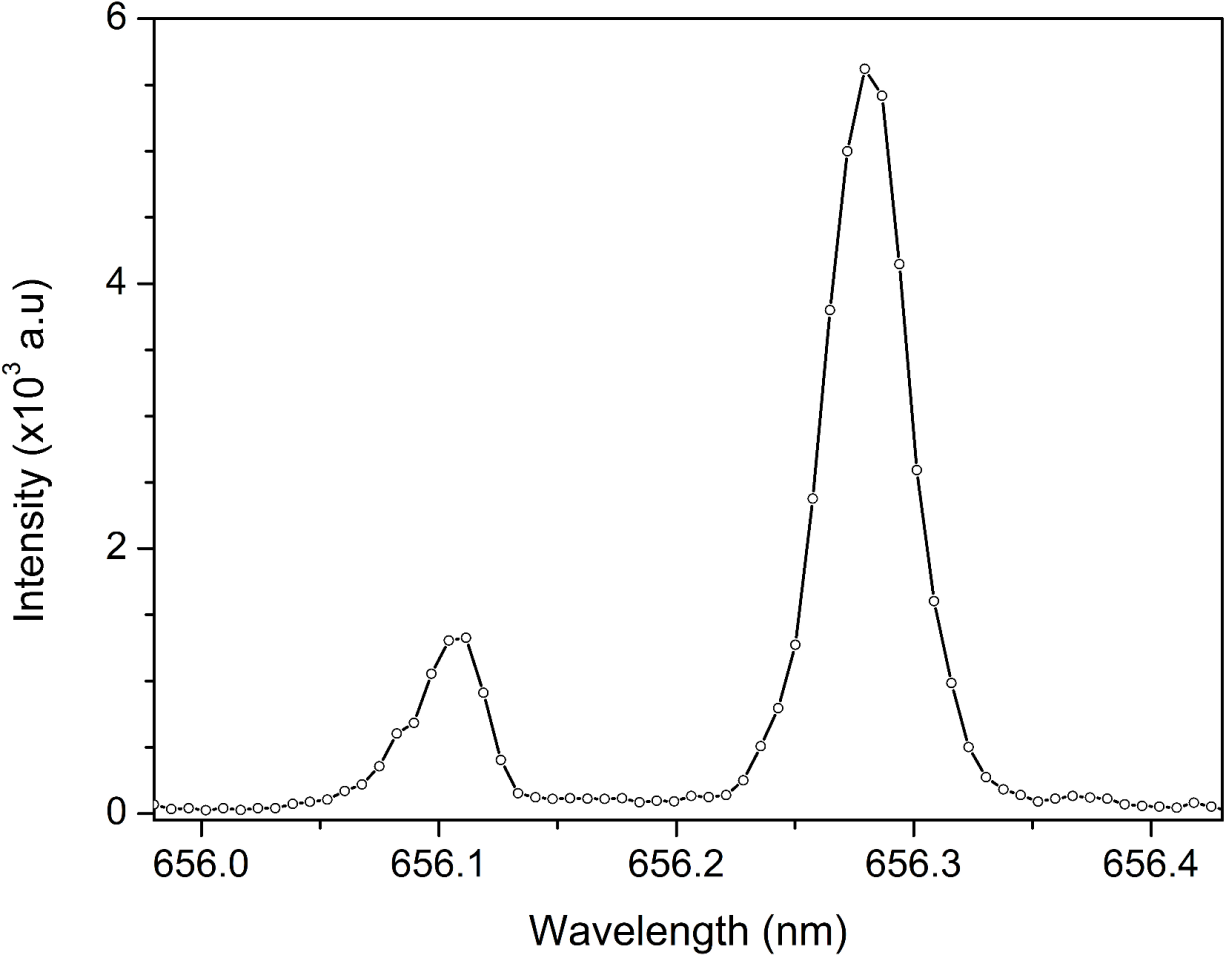
D_α_ and H_α_ lines from MIP when the vacuum pump is the sole contributor to the sample’s desorption.

Due to the composition of the target pill, water and heavy water were not fully bonded to the graphite, resulting in their evaporation from the target during vacuum pump outgassing. The corresponding MIP spectra with deuterium and hydrogen Balmer alpha lines is shown in Fig. 7. As can be seen, the lines are narrow and fully resolved.

#### Laser induced desorption

**Fig. 8:**
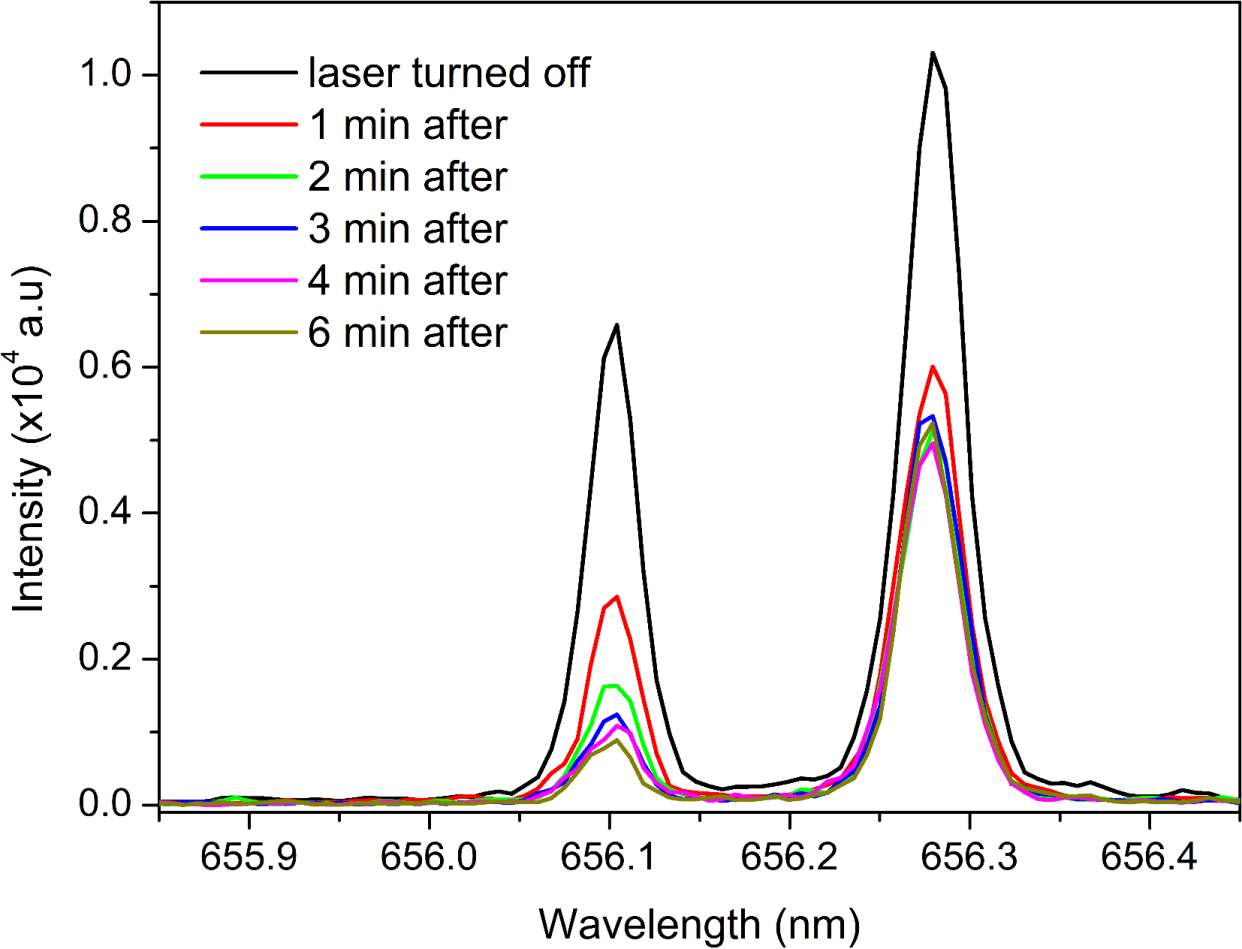
D_α_ and H_α_ line intensities after heating the target with the fs laser in the second cycle.

As a final step, we examined whether a 1030 nm fs laser with very low energy (100 nJ) could induce desorption of hydrogen isotopes from the target and introduce them into MIP. Two cycles of heating were performed and recorded. During target heating, an increase in the D_α_ and H_α_ line intensities was observed, without a corresponding increase in their FWHMs. MIP spectra, after turning off the laser following the second heating cycle, is shown in Fig. 8. The effect of laser induced desorption is evident: During the cooling of the target, the line intensities gradually decrease to the levels seen in Fig. 7, where desorption was induced by the vacuum pump alone.

**Fig. 9:**
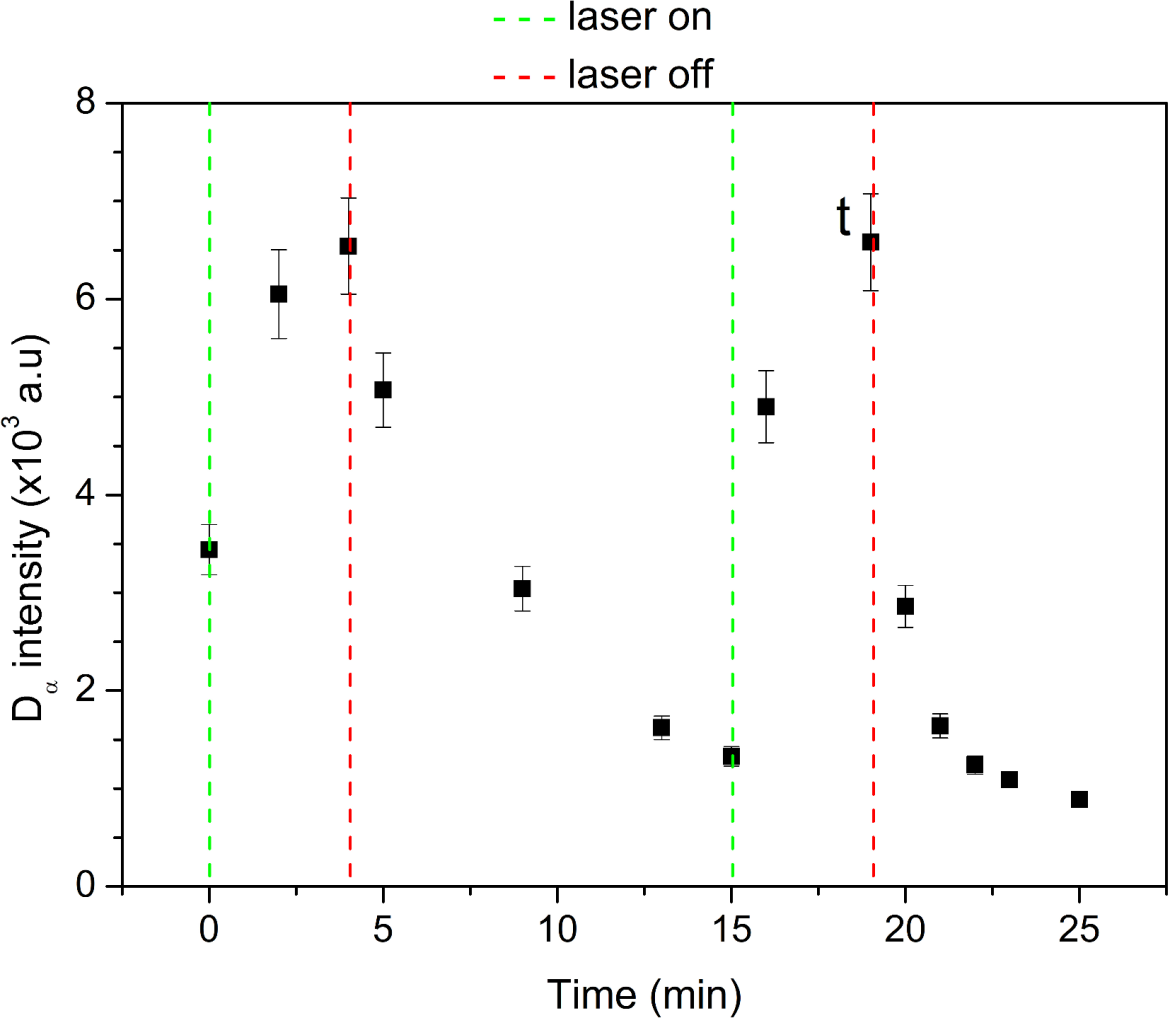
Time evolution of the D_α_ line intensity during two cycles of heating and cooling of the target.

As can be noted from Figs. 7 and 8 (6 min after), the ratio of D_α_ and H_α_ peaks remained the same after laser irradiation, but for other measurements that ratio varies. The target was made with equal shares of D_2_O (> 99% purity) and H_2_O, but there is additional contribution to the H_α_ peak that comes from the desorption of water vapor from the walls of the discharge tube. The time dependency of the D_α_ line intensity during both cycles is shown in Fig. 9. The effects of laser heating and laser induced desorption are clear. The intensity increased while the laser was active, peaking at the moments when the laser was turned off. After that, intensity decreased gradually. This confirms that the deuterium comes from laser induced desorption, rather than solely from outgassing due to the vacuum pump. It should be noted that the intensity from the first measurement is higher than it should be, because not enough time has passed for the target to cool from the previous test measurements. The starting value should be close to the one shown in Fig. 7, as that was recorded before the laser heating.

**Fig. 10:**
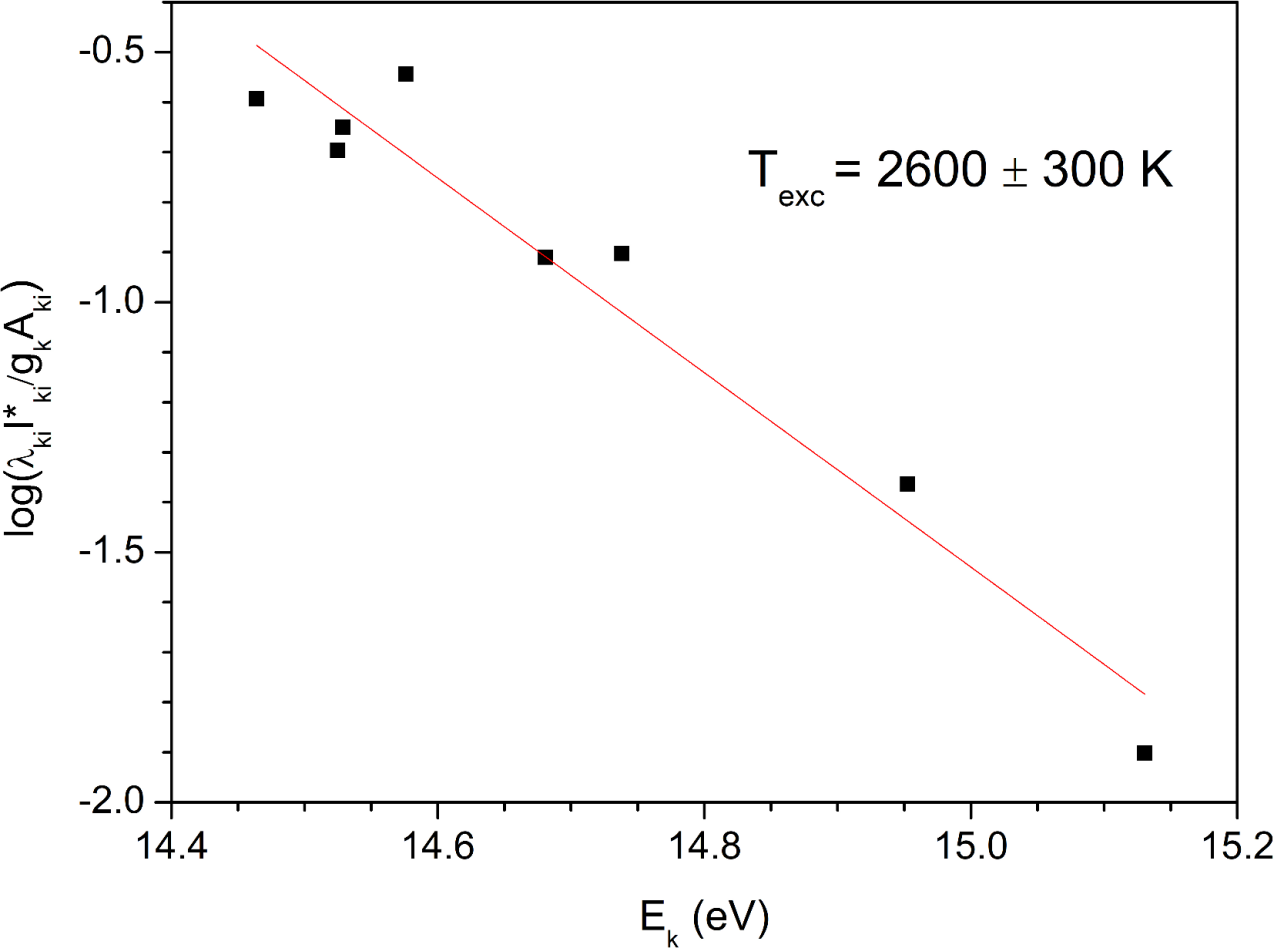
Boltzmann plot of the Ar I lines for excitation temperature estimation.

Before proceeding to the estimation of hydrogen isotope line widths, plasma parameters were estimated. The excitation temperature was estimated from the Boltzmann plot of Ar I lines to be 2600 K. Boltzmann plot is given in Fig. 10. Complete data for the lines used to obtain the Boltzmann plot can be found in the Supplementary Table S1.

**Fig. 11:**
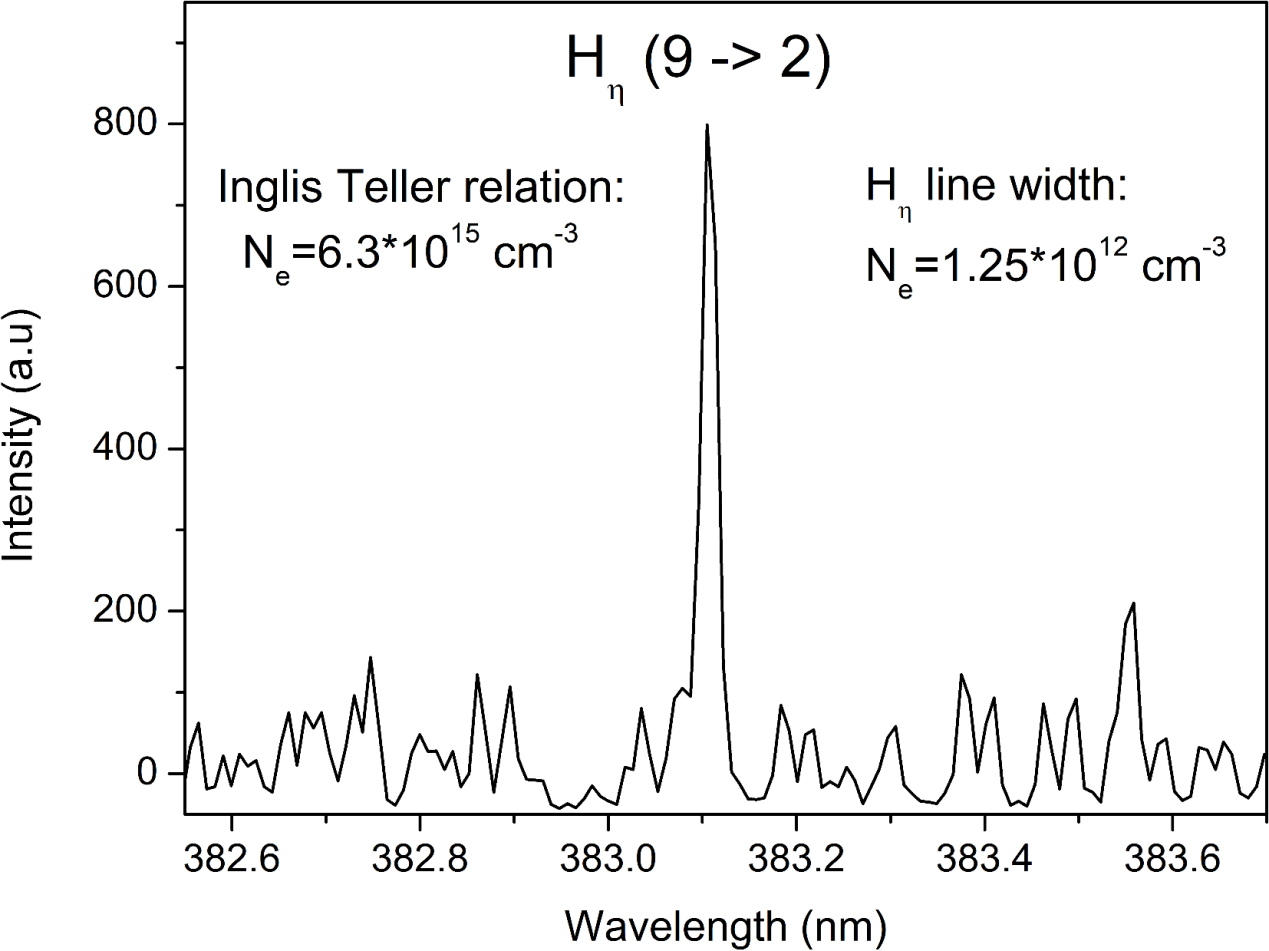
Balmer Hη line at 383.5 nm recorded for estimation of electron density.

Given the expected low electron density and negligible Stark broadening for both hydrogen and argon lines, an attempt was made to estimate the upper limit for electron density. The merging of spectral lines of hydrogen is a suitable method for this estimation. If the final detectable spectral line of Balmer series is found, then the use of Inglis – Teller relation^49^ can give the upper limit on the electron density. The final observed member of the Balmer series in this study was H-η (9 → 2, 383.5 nm), shown in Fig. 11. Assuming *N*_*e*_ = *N*_*i*_, and that for hydrogen atoms the effective nuclear charge (z) is 1, the upper limit of electron density *Ne* ~ 6.3 ∙ 10^15^ cm^−3^ was obtained. This value is a huge overestimation, since the upper members of the series couldn’t be detected due to the presence of different molecular bands. For precise resolution of superimposed signals, whether due to electric noise or molecular bands, more detailed measurements with even higher spectral resolution would need to be conducted. Regarding Balmer alpha lines, molecular bands close to them have negligible intensities, if they are present at all, and they have no influence on H_α_/D_α_/T_α_ intensity ratio.

1 $$\text{log}({N}_{e}+{N}_{i}) = 23.26-7.5\text{log}{n}_{max}+4.5\text{log}z$$

Another approach for determining the electron density is through the Stark broadening of the upper members of the Balmer series. Since the highest detected member of the Balmer series has the width close to the instrumental width, and considering the errors during the fitting procedure, it can be estimated that the Stark width of the Balmer alpha lines doesn’t exceed 0.01 nm. If this value is inserted into the formula for the estimation of line widths from the higher members of the Balmer series^50^

2 $${N}_{e}={8\cdot 10}^{18}\cdot {\left(\frac{{w}_{S} \left(nm\right)}{{\alpha }_{1/2}^{n}}\right)}^{1.5}$$

the resulting value, with *α*^*n*^_*1/2*_ = 0.345 (for *n* = 9), would be *N*_*e*_ = 1.25 ∙ 10^12^ cm^−3^, which is a more realistic estimation than the one obtained by the Inglis – Teller equation.

**Fig. 12:**
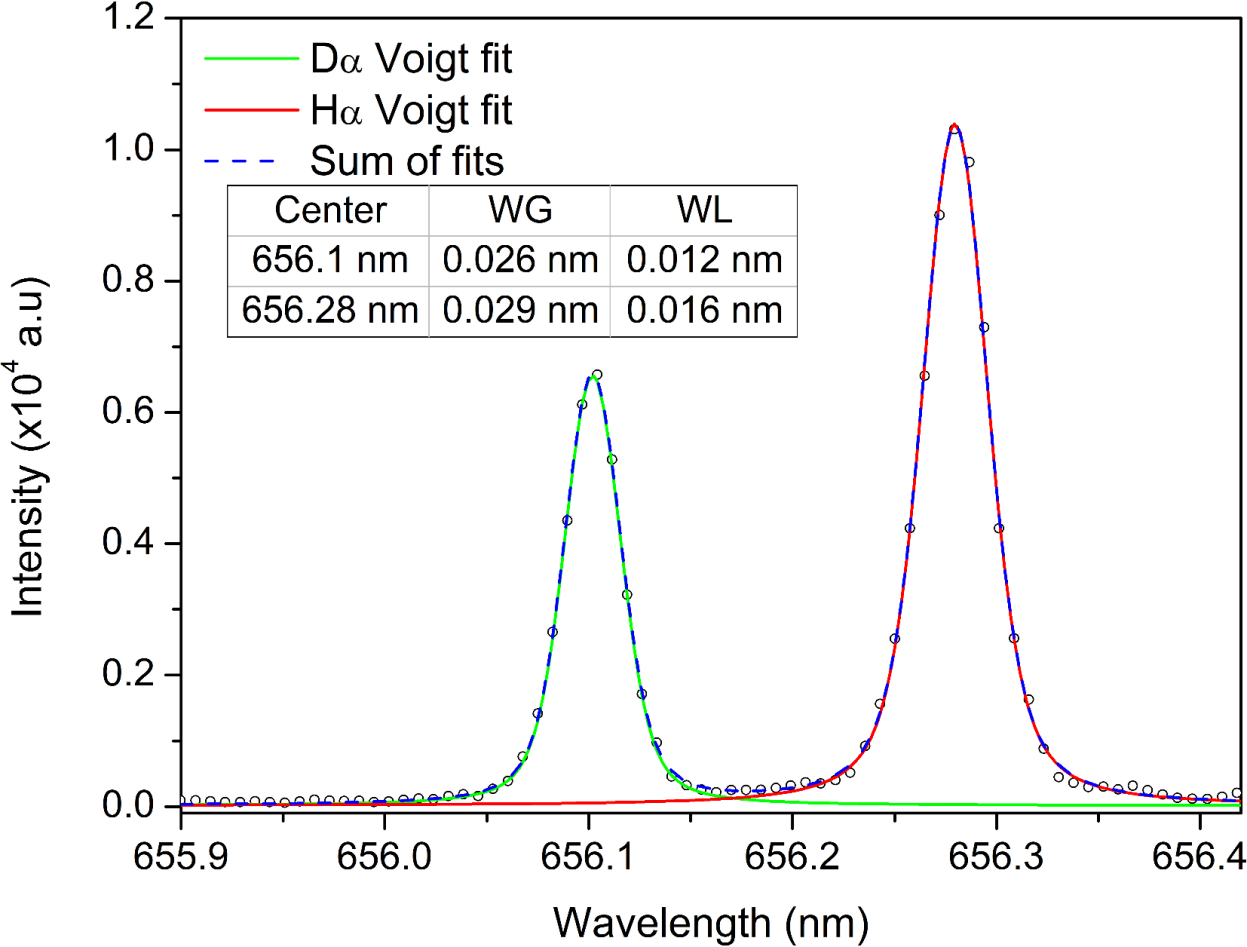
Voigt profile fitting of the D_α_ and H_α_ lines for the measurement at time t, marked in Fig. 9.

Finally, to confirm the necessary resolution for the hydrogen retention studies, both lines were fitted with a Voigt profile (Fig. 12). Gaussian fitting was also attempted, but Voigt profile showed better performance in terms of wing fitting.

Approximative equation for Voigt profile FWHM^51^

3 $${W}_{V}\approx 0.5346{W}_{L}+\sqrt{0.2166{W}_{L}^{2}+{W}_{G}^{2}}$$

was used with coefficients obtained from Fig. 12. The resulting line widths are 0.033 nm for D_α_ line and 0.038 nm H_α_ line. Although Voigt line widths are correct, the Gaussian and Lorentzian parts are off. Gaussian parts can be calculated using the equation

4 $${W}_{G}=\sqrt{\left({W}_{D}^{2}+{W}_{I}^{2}\right)},$$

where ${W}_{D}$ represents the Doppler line width and ${W}_{I}$ is the instrumental line width. Doppler broadening FWHM can be calculated using the equation

5 $${W}_{D}=7.16*{10}^{-7}\lambda \sqrt{\frac{T}{M}} ,$$

where *M* is the mass of the emitter, given in atomic mass units, and *T* is the temperature estimated using a Boltzmann plot of the Ar I lines (Fig. 10). Since the instrumental FWHM was estimated at 0.024 nm, based on FWHM of W I 429.46 nm line from Fig. 6, Gaussian parts of the Voigt profiles are 0.029 nm and 0.034 nm for D_α_ and H_α_, respectively. Lorentzian parts are then calculated using the Eq. (3), and they are 0.007 nm for both D_α_ and H_α_.

Finally, using the formula suggested in^22^, the intensity ratio of T_α_ and D_α_ for which both lines could be resolved was obtained. The critical FWHM at which mentioned lines can be resolved relates to the intensity ratio *R* = T_α_/D_α_ through the following formula^22^:

6 $$FWH{M}_{cr}=0.0599-0.0388\times {e}^{-1.765\times R}$$

Now, for the determined FWHM of D_α_ 0.033 nm, the theoretical ratio of lines is *R* = 0.2. Therefore, our proposed method could resolve D_α_ and T_α_ lines up to the point where D_α_ is five times more intense than T_α_, or vice versa.

## Conclusion

In this work, we explored the coupling of laser-induced desorption and laser ablation with microwave-induced plasma as an effective method for studying hydrogen isotope retention in plasma-facing components of fusion devices. The experimental setup was optimized to achieve high spectral resolution, demonstrating great separation of hydrogen and deuterium Balmer alpha lines, which would theoretically enable the separation of deuterium and tritium Balmer alpha lines up to the point where D_α_ is five times more intense than T_α_, or vice versa. The application of femtosecond laser for desorption facilitated sample introduction into the MIP, as evidenced by the clear enhancement of the D_α_ line. Under low-pressure MIP conditions, line broadening effects were minimized, enabling precise isotopic analysis. It should be stressed that stoichiometry problem^52^, i.e., whether the H/D ratio in the plasma corresponds to the H/D ratio in the target is under study.

This study presents a significant advancement in the diagnostics of tritium retention, offering a minimally invasive, high-resolution approach that addresses the limitations of traditional methods such as ion beam analysis and thermal desorption spectroscopy. The findings underscore the potential of this technique for in-situ applications in fusion research, contributing to the development of safer and more efficient plasma diagnostics systems. Future work will focus on scaling this approach for broader fusion reactor applications and extending it to analyze mixed material deposits.

Currently, this approach is suitable for post-mortem analysis (between experimental campaigns). The authors are working on a measuring device that will enable the application of this method for real-time in situ measurements (without demounting the components in tokamak).

## Supplementary Information

Supplementary Information.

## Data Availability

The data that supports the findings of this study is available from the corresponding author upon reasonable request.
