# Supplementary material for: Hydrogen isotopes retention studies using laser and microwave induced plasma coupling
Source: Sci Rep. 2025 Apr 12;15:12589. doi: 10.1038/s41598-025-96546-x (PMC11993670; doi:10.1038/s41598-025-96546-x)
Supplement: Supplementary file 1 — Supplementary Information. [file 41598_2025_96546_MOESM1_ESM.pdf]

Hydrogen isotopes retention studies using laser and microwave induced plasma coupling

N. Vujadinovic[1], I. Traparic[1], B.D. Stankov[1], D. Rankovic[2,3], M. Kuzmanovic[2] and M. Ivkovic[1*]

[1]*Institute of Physics, University of Belgrade, 11080 Belgrade, Serbia*

[2]*Faculty of Physical Chemistry, University of Belgrade, 11158 Belgrade, Serbia*

[3]*Present address: Vinca Institute of Nuclear Science, University of Belgrade, 11000 Belgrade, Serbia*

[*]*Corresponding author: E-mail address: ivke@ipb.ac.rs (M. Ivkovic)*

Supplementary material

Supplementary Table S1. Ar I lines data used for Boltzmann plot [1]

transition (lower – upper level)	wavelength λ_{ki} (nm)	$g_k A_{ki}$ (s^{-1})	upper level energy E_k (eV)
$3s^2 3p^5(^2P^\circ_{3/2})4s$ - $3s^2 3p^5(^2P^\circ_{3/2})5p$	416.4180	8.7e+5	14.5249
$3s^2 3p^5(^2P^\circ_{1/2})4s$ - $3s^2 3p^5(^2P^\circ_{1/2})5p$	419.1029	1.6e+6	14.6807
$3s^2 3p^5(^2P^\circ_{3/2})4s$ - $3s^2 3p^5(^2P^\circ_{3/2})5p$	419.8317	2.57e+6	14.5759
$3s^2 3p^5(^2P^\circ_{3/2})4s$ - $3s^2 3p^5(^2P^\circ_{3/2})5p$	425.1185	3.33e+5	14.4640
$3s^2 3p^5(^2P^\circ_{1/2})4s$ - $3s^2 3p^5(^2P^\circ_{1/2})5p$	425.9362	3.98e+6	14.7381
$3s^2 3p^5(^2P^\circ_{3/2})4s$ - $3s^2 3p^5(^2P^\circ_{3/2})5p$	426.6286	1.6e+6	14.5289
$3s^2 3p^5(^2P^\circ_{3/2})4p$ - $3s^2 3p^5(^2P^\circ_{3/2})5d$	603.2127	2.21e+7	15.1305
$3s^2 3p^5(^2P^\circ_{3/2})4p$ - $3s^2 3p^5(^2P^\circ_{1/2})4d$	605.9372	2.1e+6	14.9526

Reference

[1] NIST Atomic Spectra Database: https://www.nist.gov/pml/atomic-spectra-database (last update to data content: November 2024).
